# Generation of functional neurons from adult human mucosal olfactory ensheathing glia by direct lineage conversion

**DOI:** 10.1038/s41419-024-06862-9

**Published:** 2024-07-03

**Authors:** María Portela-Lomba, Diana Simón, Marta Callejo-Móstoles, Gemma de la Fuente, David Fernández de Sevilla, Vega García-Escudero, M. Teresa Moreno-Flores, Javier Sierra

**Affiliations:** 1 School of Experimental Sciences, Universidad Francisco de Vitoria, Pozuelo de Alarcón, Spain; 2 Department of Anatomy, Histology and Neuroscience, School of Medicine, Universidad Autónoma de Madrid, Madrid, Spain; 3 School of Medicine, Universidad Francisco de Vitoria, Pozuelo de Alarcón, Spain; 4 Present Address: Department of Genetics and Development, Columbia University Medical Center, New York, NY USA

**Keywords:** Regeneration and repair in the nervous system, Stem-cell research

## Abstract

A recent approach to promote central nervous system (CNS) regeneration after injury or disease is direct conversion of somatic cells to neurons. This is achieved by transduction of viral vectors that express neurogenic transcription factors. In this work we propose adult human mucosal olfactory ensheathing glia (hmOEG) as a candidate for direct reprogramming to neurons due to its accessibility and to its well-characterized neuroregenerative capacity. After induction of hmOEG with the single neurogenic transcription factor NEUROD1, the cells under study exhibited morphological and immunolabeling neuronal features, fired action potentials and expressed glutamatergic and GABAergic markers. In addition, after engraftment of transduced hmOEG cells in the mouse hippocampus, these cells showed specific neuronal labeling. Thereby, if we add to the neuroregenerative capacity of hmOEG cultures the conversion to neurons of a fraction of their population through reprogramming techniques, the engraftment of hmOEG and hmOEG-induced neurons could be a procedure to enhance neural repair after central nervous system injury.

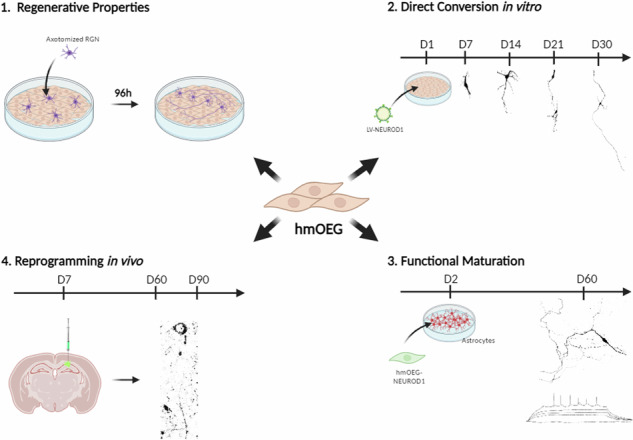

## Introduction

Since Ramon y Cajal classical studies it is known the scarce regenerative capacity of the Central Nervous System (CNS) [1]. A recent approach to foster CNS regeneration after injury or disease is cellular direct conversion: to reprogram somatic cells into terminally differentiated neurons without going through a stem cell stage [2–4]. This is achieved by virus-mediated ectopic expression of neurogenic transcription factors on their own or in combination with other factors to enhance cell maturation, efficiency or specification [3, 5]. The induced neurons (iNs) are either generated in vitro and subsequently transplanted at the site of the lesion or reprogramming is induced from glial cells located at the affected area [6]. This strategy bypasses the reported inconveniences associated with induced pluripotent stem cells (iPSCs) for cell replacement therapy: tumorigenesis, indeterminate differentiation or genomic instability [7, 8].

The choice of the cell type to be reprogrammed is a major challenge for the potential applications of iNs. Fibroblasts are an abundant and easily accessible cell type and have been successfully converted into various neuronal subtypes, even though it is a cell lineage distantly related to neurons [9–11]. Astrocytes are one of the main cell sources for direct conversion to neurons as they share a common neural origin and they are ubiquitously distributed throughout the CNS. Thus, it has been reported that expression of neurogenic transcription factors NEUROG2 or ASCL1 in postnatal mouse astrocytes generates glutamatergic or GABAergic neurons [12, 13]. In vivo astrocyte to glutamatergic neuron conversion has also been accomplished by expression of a single neural transcription factor, NEUROD1 [14, 15] or striatal astrocytes into GABAergic neurons through ectopic expression of NEUROD1 and DLX2 transcription factors [16]. Other glial cells such as microglia and NG2 have also been reprogrammed to neurons using the NEUROD1 factor [17] or combining three factors - ASCL1, LMX1, NURR1 - [18, 19], respectively. Nevertheless, these glial cell types are difficult to obtain from patients.

In this work we selected olfactory ensheathing glia (OEG) as a candidate for direct conversion to neurons. An advantage of OEG over other cell types is the reported capacity of OEG in promoting CNS regeneration [20]. In adult lifetime, olfactory sensory neurons are constantly renewed and OEG is responsible for facilitating axonal growth from the neuroepithelium to mitral and tufted cells in the olfactory bulb [20, 21]. OEG neuroregenerative capacity, both from olfactory bulb and mucosa, has been tested in an in vitro model of axotomized rat retinal ganglion neurons [22–24] and in vivo in rat models of spinal cord injury (SCI) [25–28]. Its reparative ability is due to a combination of several factors: these cells express membrane bound and secreted adhesion molecules that promote axonal growth [20], they secrete proteases [29, 30] being able to decrease reactivity and size of the glial scar [31–33] and they secrete neurotrophic factors [34, 35] and cytokines [21] that play a relevant role in neuroprotection and repair in the damaged zone. Furthermore, these cells can easily be obtained from the olfactory mucosa of patients by biopsy [22] so autologous therapies can be performed and avoid post-transplant rejections.

Thus, if we add to the neuroregenerative capacity of OEG cultures the conversion to neurons of a fraction of their population through reprogramming techniques, the engraftment of OEG and OEG-induced neurons (OEG-iNs) could enhance neural repair at the damaged site. In the present work, we characterized a primary OEG culture from adult human olfactory mucosa (hmOEG) that showed neuroregenerative properties. After transduction of the single neurogenic transcription factor NEUROD1, the cells under study not only exhibited morphological and immunolabeling neuronal features but were able to fire action potentials and expressed glutamatergic and GABAergic markers. In addition, after engraftment of transduced hmOEG cells in mouse hippocampus, these cells showed specific neuronal labeling. These findings lay the groundwork for the potential use of hmOEG-iNs in cell-based therapy or disease modeling.

## Materials and methods

### Animals

All animal procedures were carried out complying with the European Council Directive 2010/63/UE and Spain RD 53/2013, and approved by national and institutional bioethics committees with the authorization code PROEX 142.2/20. Animals were housed under a 12-hour light/12-hour dark cycle and were supplied with ad libitum access to regular food and water.

Animal procedures were performed in animal facilities of Universidad Francisco de Vitoria and Facultad de Medicina of Universidad Autónoma de Madrid. The surgeries of the NOD-SCID mice were carried out in the Instituto de Investigaciones Biomédicas (IIBm-CSIC-UAM) facilities under a flow laminar hood.

Adult male Wistar rats (RccHan®:WIST) were obtained from Envigo (Envigo RMS Spain, SL). We used a total of four Wistar rats for hmOEG – retinal neurons coculture studies. Wild-type C57BL6 mice were obtained from the animal facility of Universidad Francisco de Vitoria, and we used a total of 20 C57BL6 mice (10 postnatal of 2 days, and 1 adult pregnant female mouse to extract a total of 9 embryos of 18 days) for neuronal and astrocyte cultures. Immunosupressed NOD-SCID mice were acquired and maintained in the Instituto de Investigaciones Biomedicas “Alberto Sols” (IIBM) animal facility under sterile conditions. We used a total of 12 NOD-SCID mice for hmOEG transplantation (4 animals/group × 3 survival times, 1, 2 and 3 months). Animals were randomly allocated to each experimental group without further randomization methods, and none was excluded from the study. Based in our previous experience with cell transplantation, we calculated a minimum number of animals to obtain consistent results while adhering to the 3Rs. MP-L, MC-M and MTM-F oversaw and knew group allocation.

### hOEG cell cultures

Primary cultures of human olfactory mucosa (hmOEG) were derived from a human nasal endoscopic biopsy of a 30- year-old patient (woman) carried out during a septoplasty at the Department of Otolaryngology, Gregorio Marañón Hospital, Madrid [36]. hmOEG and immortalized human olfactory bulb OEG (ihOEG) cell lines Ts14 [37, 38] and Ts12 [39], were maintained in ME media, composed by DMEM/F12 (Gibco) supplemented with 10% FBS (GE Healthcare Hyclone), 2 mM glutamine (Lonza), 20 µg/mL pituitary extract (Gibco), 2 μM forskolin (FSK; Sigma) and antibiotics (P/S/A, penicilin/estreptomicin/anfotericin; Lonza) at 37 °C in 5% CO_2_.

### Mouse neuron primary culture

Mouse embryonic neurons were obtained from cerebral cortex of E17 C57BL6 mice using the Worthington Biochemical Corporation papain dissociation kit (ref. LK003150). After removing the meninges, the cortex was transferred to a papain plate, where they were cut in <1 mm pieces with a scalpel blade. Then, they were incubated at 37 °C for 30 min with intermittent shaking. Afterwards, they were mechanically disaggregated by pipetting with a glass Pasteur 10–15 times until a homogeneous suspension was obtained. Subsequently, in order to remove the remained aggregates, the suspension was passed through a 0.75 mm mesh and then centrifuged for 5 min at 200 × *g*. Supernatant was aspirated and cells were resuspended in 5 mL of NB-B27 Plus medium (Neurobasal Plus (NB, Gibco) supplemented with B27 Plus (Gibco), 2 mM glutamine (Lonza) and P/S/A (Lonza)) and 100,000 cells were plated on glass coverslips (12 mm, ThermoScientific) pre-treated with PLL-Laminin (5-10 µg/mL).

### Mouse astrocyte primary culture

Astrocytes were isolated from the cortex of postnatal 0-2 days old (P0-2) C57BL6 mice. Cerebral cortex was dissected and, after removal of meninges, cut with a scalpel blade until <1 mm pieces were obtained. Then, they were passed through a 5 mL pipette 5 times and incubated at 37 °C for 30 min with intermittent shaking. Afterwards, they were mechanically disaggregated by pipetting with a glass Pasteur 20–25 times until a homogeneous suspension was obtained. Subsequently, the suspension was passed through a 0.75 mm mesh to remove clumps and centrifuged for 5 min at 200 × *g*. Thereafter, the supernatant was aspirated, cells were resuspended in the corresponding volume of M10 medium (1 mL per 4 hemispheres) composed of DMEM/F12 (Gibco) supplemented with 10% FBS (GE Healthcare Hyclone), 2 mM glutamine (Lonza) and P/S/A (Lonza), and 1 mL of the supernatant was plated in a T75 culture flask (hereafter T75 flask; Falcon), pre-treated with poly-L-lysine (PLL, 10 µg/mL; Sigma). When the cells reached 90–95% confluence, around day 7–10 post-cultured, the T75 flasks were shaken O/N at 37 °C to obtain a purified astrocyte culture.

### hOEG co-cultures with retinal ganglion neurons (RGNs)

Cocultures of hOEG with axotomized RGNs were carried out to assess the neurogenerative capacity of hOEG as previously described [24]. Briefly, hOEG cells were plated on 12 mm coverslips in a 24-well plate (M24; Cultek), aiming to have a monolayer the following day. RGNs were axotomized and extracted from retinas of 2-month-old adult rats (Wistar rats; Envigo) by sectioning the optic nerves and were dissociated using the papain kit from Worthington Biochemical Corporation. Isolated RGNs were plated on top of the hOEG monolayers and after 96 h in coculture, these were fixed with 4% paraformaldehyde (PFA) for immunostaining.

To evaluate the regenerative capacity of hOEG over the axotomized RGNs, the cocultures were immunolabeled with SMI31 (MAP1B and neurofilament H axonal markers) and MAP2A&B (somatodendritic marker) and analyzed with the 40× objective of an inverted fluorescent microscope (DMi8, Leica). Immunofluorescence images were quantified with ImageJ software (ImageJ; NIH) and axon length was measured using the NeuronJ plugin. Quantification of axonal regeneration was determined by calculating: (1) the percentage of neurons with axons, detected with SMI31, versus the total number of neurons, labeled with MAP2A&B; (2) the mean axonal length per neuron, by calculating the ratio of the sum of the lengths (µm) of all axons out of the total number of neurons counted. At least 30 fields were randomly imaged and at least 200 neurons were quantified in each preparation. Experiments were repeated independently at least three times.

### Lentivirus production

The packaging plasmids pMD2.G (#12259) and PAX2 (#12260) were acquired from Addgene. The plasmids pCAG-*Gfp*-IRES-*Gfp* (LV-*Gfp*) and pCAG-*NeuroD1*-IRES-*Gfp* (LV-*NeuroD1*) were obtained in retroviral vectors, kindly donated by Dr. Gong Chen [15] and then they were cloned into pRRL.sin.cPPT.CMV.Wpre [40]. pCAG-*Ngn2*-IRES-GFP (LV-*Ngn2*) [41] was kindly supplied by Dr. Guillermina López-Bendito. *Ascl1* was cloned into pLenti CMV/TO Neo empty (w215-1) plasmid (Addgene #17485).

For the lentiviral production, 5×10^6^ HEK293T cells were plated in 10-cm plates (p100, Falcon). The following day, cotransfection of 5 µg of pMD2G, 6 µg of PAX2 and 10 µg of the lentiviral vector were carried out using the calcium phosphate method [42] in Optimem media (Gibco). After 6 h post transfection, a 10% glicerol shock was performed. First, a solution composed by glicerol:HBS:ddH_2_O (1:5:4) was prepared. Then, the media was aspirated and 2 mL of the glicerol solution was added to each p100 during exactly 2 min. Subsequently, the solution was aspirated and washed with PBS 1× before adding 7 mL of M10 media per plate. After 72 h post transfection, the media containing the lentiviral particles was harvested, filtered through a low binding 0.45 µm mesh and concentrated at 10 °C in a ultracentrifuge (OPTIMA XE-90) for 2 h at 82,700 × *g*. Finally, the pellet containing the virus was resuspended in PBS 1× and stored at −80 °C. Viral stocks were titered by flow cytometry, after infection of HEK293T cells, to get the % of GFP+ cells. Titer average was 10^7^ transducing units (TU) /ml.

### hmOEG reprogramming

For neuronal reprogramming, 20,000 cells/well of hmOEG were plated in M24 cell plates (M24-I, Ibidi) pre-treated with poly-L-ornitine/laminin (PO/L: 20 µg/mL/5 µg/mL) to reach 80-90% of confluence. Next day, cells were infected with the lentiviral particles in M10 media containing polybrene (8 µg/mL, Sigma) with a MOI of 10. After 6–20 h, the viral media was changed with fresh M10 medium. The following day, the media was replaced with neuronal diferentiation media 1 (NDM1: DMEM/F12 (Gibco), glutamax (Gibco), P/S/A (Lonza), glucose 3.5 mM (Sigma), FBS 1% (Gibco), N2 (Gibco) and B27 (Gibco)) with 10 μM FSK. Media was partially renewed every 3-4 days with neuronal differentiation media 2 (NDM2: 1:1 DMEM/F12:NB (Gibco), glutamax (Gibco), P/S/A (Lonza), glucose 3.5 mM (Sigma), N2 (Gibco), B27 (Gibco) and 20 ng/mL of the maturation factors BDNF, GDNF and NT3) containing 10 μM FSK.

### hmOEG-induced neurons (hmOEG- iNs) coculture with astrocytes

Infected hmOEG were cultured onto a monolayer of mouse postnatal astrocytes to increase hmOEG-iNs viability. For the coculture, 75.000 astrocytes were plated in M24-I pretreated with polyornitine (20 µg/mL)–laminin (5 µg/mL). The remaining astrocytes were plated in 150 mm plates (P150), pretreated with PLL to obtain conditioned NDM2 from the astrocytes. After 48 h, 20,000 hmOEG-iNs (48 h post infection) were plated onto the astrocytes in ME media. Next day, the medium was replaced by conditioned NDM1 with 10 μM FSK. Every 3–4 days the media was refreshed by conditioned NDM2 containing 10 μM FSK. The cocultures were maintained up to 90 days.

### Cell transplantation

NOD/SCID mice 1 month old were anesthetized with isoflurane. Transduced hmOEG (7 days post infection) were stereotactically transplanted at a concentration of 200,000 cells/2 µL (infusion rate of 0.5 µL/min), into the hippocampus (right hemisphere) in the following coordinates from Bregma: AP-2, ML1.5/-1.5, DV-2. Mice were sacrificed 2 or 3 months post injection for immunohistological analysis.

### Immunostaining

Cell cultures were fixed with 4% PFA, permeabilized and blocked with PBS-TS (PBS, 0.1% Triton, 5% FBS) for 30 min at RT and then incubated at 4 °C O/N with the corresponding primary antibody diluted in PBS-TS. Next day, three 5-min washes were performed with 1× PBS and incubated with the desired fluorescent secondary antibody (Alexa), diluted in PBS-TS, for 1 h at RT in the dark. Afterwards, they were washed 3 times with 1× PBS for 5 min and incubated with DAPI (4′6-Diamidino-2-phenylindole; 1 µg/mL, MERCK) for 5 minutes. After washing, samples were mounted with fluoromount (Southern Biotech) and observed under an inverted fluorescent microscope (DMi8, Leica). Antibodies and antibodies dilutions are listed in Supplementary Table 1. Fields were randomly photographed, and the intensity density of the somas was quantified in an automated and blind way, taking as region of interest the DAPI staining. Immunofluorescence signals were quantified using ImageJ software (ImageJ; NIH).

For the cerebral tissues, mice were anesthetized with 0.2 mL of pentobarbital (33 mg/mL) and then intracardially perfused with 4% PFA. Subsequently, brains were extracted, embedded in *Tissue-Tek OCT Compound*, and sliced in the cryostat at 25 µm. For the immunohistochemistry, the brain slices were blocked with PBS-TS2 (PBS, 0.5% Triton, 10% serum and 1% BSA) for 1 h at RT and then incubated with the desired primary antibody at 4 °C O/N. Next day, the brain slices were washed 3 times with PBS for 10 min before the incubation with the fluorescent secondary antibody (Alexa) for 2 h at RT in the dark. Then, three 10-min washes were performed with PBS, following an incubation with DAPI for 10 min. After washing, brain slices were placed in slides, dehydrated and mounted with DEPEX (MERCK). Samples were examined with a Leica TCS SP5 spectral confocal microscope and for image acquisition, a LAS-AF software was used. Images were taken with 20× and 40× objectives and using 405 nm (DAPI staining) 488 nm (green immunostaining) and 561 nm (red immunostaining) laser lines.

### Electrophysiology

hmOEG-iNs 30-, 60- or 90 days post infection were recorded using the patch-clamp technique in their whole-cell configuration in voltage-clamp and current-clamp modes. Recordings were made with borosilicate pipettes (OD-ID: 1.5–0.86; Sutter Instrument CO, Novato, CA), with a resistance of 4–8 MΩ and were filled with an intracellular solution (5 mM KCl, 20 mM HEPES, 2 mM CaCl2, 2 mM MgCl2, 0.6 mM EGTA, 130 mM K-gluconate, 2 mM ATP, 0.2 mM GTP, 20 mM phosphocreatine and 50 U/mL creatine phosphokinase; pH 7.3 and osmolarity 286 mOsm). Recordings were performed at a temperature of 30 °C with a constant flow of 2 mL/minute extracellular solution (150 mM NaCl, 2.5 mM KCl, 10 mM HEPES, 30 mM glucose, 1 mM MgCl_2_ and 2 mM CaCl_2_; pH 7.3). Action potentials and sodium currents were evoked by depolarizing pulses of current and voltage. Cells were accepted only when the seal resistance was above 1 GΩ and the series resistance did not change by 20% during the experiment. Signals were filtered at 3 KHz and sampled at 10 KHz with a Digidata 1500 A analog-to-digital conversion card (Molecular Devices, Sunnyvale, CA). Mice embryonic cortex neurons cultured in vitro between 7–14 days were recorded as a positive control. After obtaining a stable recording of action potentials and sodium currents, recordings were blocked by adding tetrodotoxin (TTX; 0.5 µM, Abcam), a blocker of voltage-dependent sodium channels, to the extracellular solution.

### Statistical analysis

For statistical comparisons between two populations Student *t*-test was applied. For multiple comparisons, one way ANOVA and post hoc Tukey test was applied. To analyze the intensity density of the immunofluorescences, two-factor analysis of variance (two-way ANOVA) followed by the multiple comparison between means Sidak’s post-hoc test was applied. Statistical significance was established at a value of *p* < 0.05.

## Results

### Primary cultures from adult human olfactory ensheathing glia show neuroregenerative properties

To verify the identity of human olfactory mucosa (hmOEG) primary culture we carried out an expression analysis of OEG and neuronal markers by immunofluorescence techniques. As positive controls we used a previously described olfactory bulb immortalized human OEG cell line (TS14, from now on) [38, 39] and mouse embryonic cortex neurons. hmOEG cells stained positive for OEG markers S100 glial calcium binding protein B (S100B) and vimentin, showed the characteristic diffuse pattern for glial fibrillary acid protein (GFAP) [23, 37] (Supplementary Fig. S1), a constitutive weak cytoplasmic labeling for β3-tubulin (TUBB3) (Tuj1) [22] and lacked staining for neuronal nuclear antigen NeuN/FOX3 (Supplementary Fig. S2).

To rule out the presence of neuronal precursors we performed immunofluorescence to detect SOX2, expressed in neural progenitor cells. As a positive control we used the above-mentioned neuronal primary culture from mouse embryonic cortex, which due to its embryonic stage is a niche for neuronal precursors. Our results excluded the presence of neuronal precursors in our hmOEG cell line: the embryonic cortex culture but not hmOEG cells expressed SOX2 (Supplementary Fig. S2).

**Fig. 1:**
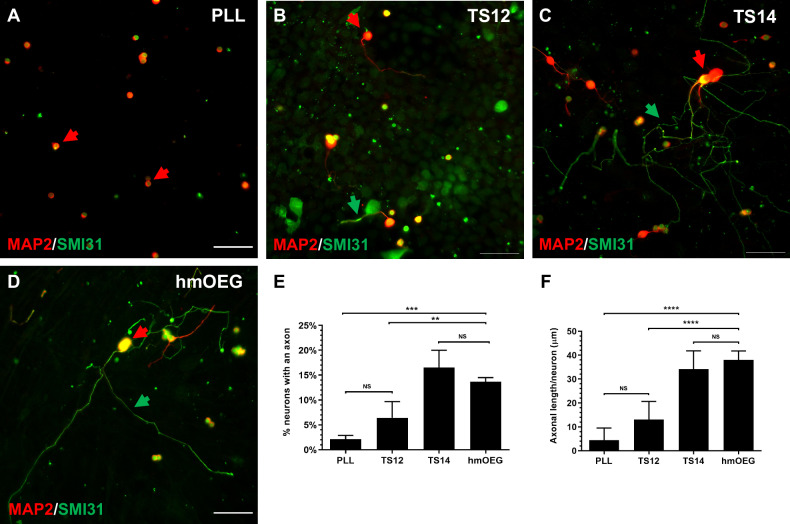
hmOEG shows regenerative capacity in an in vitro model of adult axonal regeneration. **A**–**D** Representative images of axotomized retinal ganglion neurons after 96 h in co-culture with OEG. OEG human cell line TS14 was used as a positive control for neuro-regeneration (**C**) and human cell line TS12 (**B**) and PLL (**A**) correspond to low regeneration and negative controls, respectively; (**D**) corresponds to hmOEG. Green arrows indicate axons positive for axonal marker SMI31 (green) and red arrows indicate somas and dendrites positive for somatodendritic marker MAP2 (red). Histograms represent the mean ± SD of the quantifications: percentage of retinal neurons extending an axon (**E**) and the mean axon size expressed as μm/neuron (**F**). Statistical tests applied were one-way ANOVA and post-hoc Tukey test (****p ≤ 0.0001; ***p ≤ 0.001; NS not significant) for multiple comparisons between means (*n* = 4, per experiment ≥ 30 fields were analyzed). Scale bar: 50 μm.

Next, we assessed the regenerative properties of hmOEG. For this purpose, we conducted an in vitro regeneration assay in a co-culture of hmOEG with axotomized adult rat retinal ganglion neurons (RGNs) (see Materials and Methods section). TS14, a line with regenerative capacity, and TS12, an immortalized OEG line with low regenerative capacity, were used as controls [39]; as a negative control we grew axotomized RGN cells on a poly-L-lysine (PLL) substrate to verify that they did not inherently extend axons. Axonal regeneration was quantified using immunostaining against SMI31 (a phosphoepitope of neurofilament H and MAP 1B) as an axonal marker and against MAP2A/B to identify the somatodendritic compartment. Two parameters were assessed to determine regenerative capacity: the percentage of RGNs that extended an axon and the mean axonal length/neuron of these axons. We observed that the regenerative capacity of hmOEG was similar to our positive control: percentage of neurons with an axon was 14 ± 1% and the average axonal length was 38 ± 4 micrometers/neuron (Fig. 1). Furthermore, a statistically significant higher regenerative capacity of both parameters was observed with respect to the low regenerative Ts12 and negative PLL controls (Fig. 1).

In summary, the hmOEG primary cell line expressed OEG markers but did not express neuronal or neuronal precursor markers and, moreover, showed proregenerative properties in an in vitro model of adult axonal regeneration.

### Ectopic expression of NEUROD1 directly converts hmOEG to neuronal cells

We selected the transcription factors NEUROG2, NEUROD1, and ASCL1, which are proteins of the bHLH family of transcription factors involved in specification and neural differentiation, for screening to convert hmOEG to induced neurons (hmOEG-iNs). hmOEGs were infected with lentiviral particles to express the candidate factors, individually or in combination with each other (see “Methods” section) and were identified by their co-expression with the green fluorescent protein (GFP). As a negative control we infected the cells with the same lentivector, but the transcription factor gene was replaced with a second copy of the GFP gene (LV-GFP from now on).

We assessed the reprogramming competence of the transcription factors by immunostaining with the pan neuronal marker Tuj1 in GFP-positive cells, 30 days post-infection (dpi). Ectopic expression of NEUROD1 induced Tuj1 expression and a morphological change towards a neuronal phenotype; by contrast, neither LV-GFP nor the other candidate genes triggered conversion of hmOEG into Tuj1-positive neuronal cells (Supplementary Fig. S3A–D′ and data not shown for ASCL1). Based on these results, we selected the transcription factor NEUROD1 to study the reprogramming process.

**Fig. 2:**
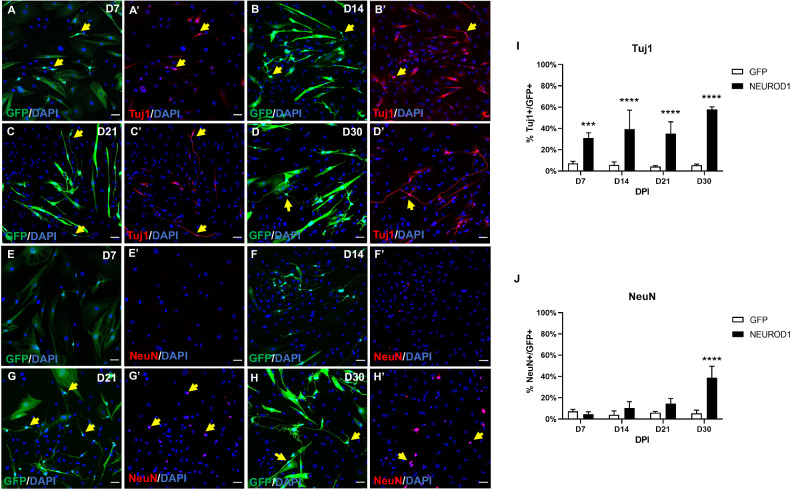
NEUROD1-hmOEG express neuronal markers. Representative images of the neuronal markers Tuj1 (**A**–**D**′) and NeuN (**E**–**H**′), expressed at 7, 14, 21, and 30 dpi. Note the neuronal morphology at 30 dpi. GFP+ cells carry the NEUROD1-GFP construct. Yellow arrows indicate cells expressing the marker. The histograms (**I**, **J**) represent the mean ± SD of the percentage of Tuj1 or NeuN positive cells in relation to the total number of infected, GFP+ cells (NEUROD1: carry the NEUROD1-GFP construct, GFP: carry the GFP-GFP construct). Statistical tests applied were Two-way ANOVA and post-hoc Sidak test (****p ≤ 0.0001,***p ≤ 0.001) for multiple comparisons between means (*n* = 3 independent experiments, 25 fields were analyzed). Scale bar: 50 µm. dpi: days post infection.

To determine the reprogramming efficiency of NEUROD1 ectopic expression, we calculated the percentage of Tuj1-positive cells in relation to the total number of infected, GFP-positive cells (Tuj1+/GFP+). At 7 dpi the number of Tuj1 expressing cells constituted 31 ± 5% of the infected cells, reaching 58 ± 2% after 30 dpi (Fig. 2A–I). In addition, representative images show a change in cell morphology along time, with a gradual development of neuronal-like extensions (Fig. 2A–D′). Immunostaining of the mature neuronal marker NeuN showed positive signal after 21 dpi (Fig. 2E–G′, J) but it was not until 30 dpi when a significant increase (39 ± 11%) of GFP-positive cells expressing NeuN was observed (Fig. 2H, H′, J).

**Fig. 3:**
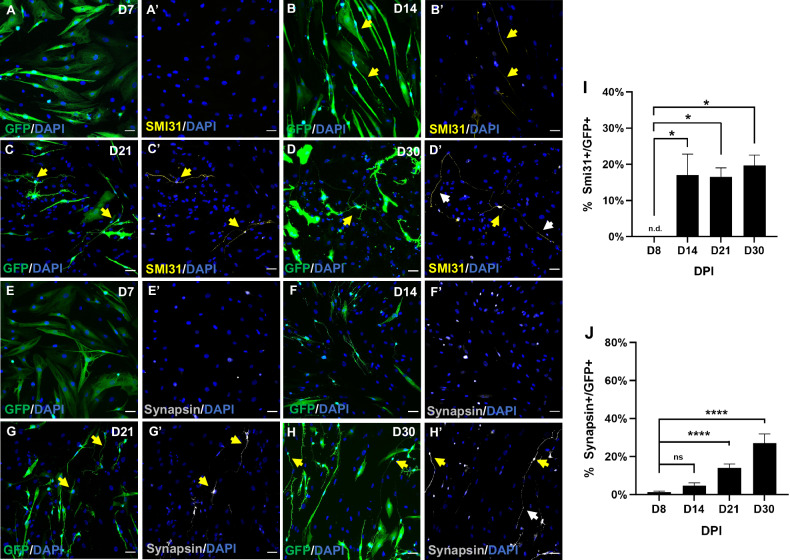
Expression analysis of mature neuronal markers in NEUROD1-hmOEG. Representative images of neuronal markers SMI31 (**A**–**D**′) and synapsin (**E**–**H**′), expressed at 7, 14, 21, and 30 dpi. Yellow arrows indicate cells expressing the marker. Of note is the pattern of SMI31 expression in a positive gradient from the soma towards the axon ends (**D**’, white arrows), a characteristic axonal pattern. Synapsin labeling along the axon is also visible (H’, white arrowheads). GFP+ cells carry the NEUROD1-GFP construct. The histograms (**I**, **J**) represent the mean ± SD of the percentage of GFP+ cells expressing SMI31 or Synapsin over the total of GFP+ cells. Statistical tests applied were Two-way ANOVA and post-hoc Sidak test (****p ≤ 0.0001,*p ≤ 0.05) for multiple comparisons between means (*n* = 3 independent experiments, 25 fields were analyzed). Scale bar: 50 µm.

We further examined neuronal maturation by immunocytochemical analysis of the axonal marker SMI31, whose expression in hmOEG-iNs began at 14 dpi and was held over time (Fig. 3A–D′). It is worth noting that at 30 dpi, hmOEG-iNs expressed SMI31 in a positive gradient from the soma towards the axon ends (Fig. 3D´), a pattern characteristic of mature neurons. Likewise, hmOEG-iNs stained positive for the presynaptic neuronal marker synapsin from 21 dpi onwards, with a characteristic expression in discrete puncta along the axon, at 30 dpi (Fig. 3E–H′).

To study whether the reprogramming process of hmOEG to neurons could pass through a neural stem cell-like state prior to differentiating into iNs [43–45] we analyzed the expression of SOX2, a characteristic marker of neuronal precursors, during the first days of induction (2, 4 and 6 dpi). In parallel, we also assessed the expression of the cell proliferation marker KI67. Immunocytochemical analysis showed that 72 ± 7% of NEUROD1 infected hmOEG were in a proliferative stage at 2 dpi, but there was a significant drop in the percentage of cells expressing KI67 at days 4 and 6 dpi (Supplemental Figure S4), suggesting that there was no expansion of progenitor cells during the hmOEG-to-neuron conversion. Regarding SOX2 expression, there was no significant variation over the days in the percentage of positive cells for this neural precursor marker (Supplemental Fig. S4), suggesting that NEUROD1-induced cells do not go through a dedifferentiation stage.

In summary, the infection of hmOEG with LV-*Neurod1* induces the acquisition of a neuronal phenotype over time, as evidenced by a neuronal morphology and the expression of mature neuronal markers, without transitioning through a neuroprogenitor condition.

### Functional maturation of hmOEG-iNs

**Fig. 4:**
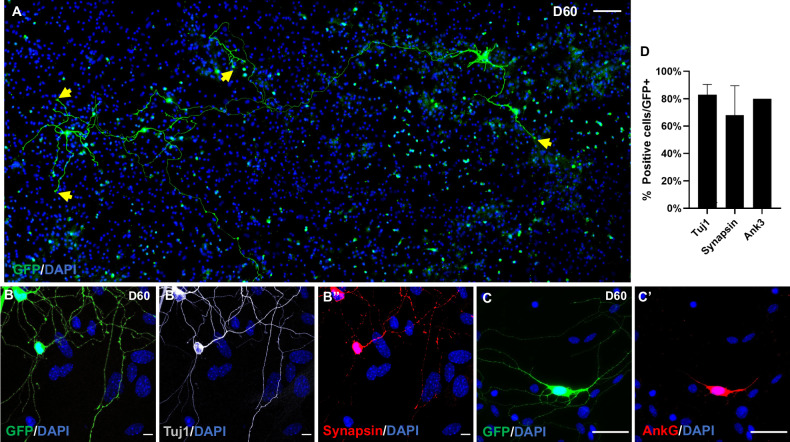
Maturation of hmOEG-iNs. **A** Tilescan of hmOEG-iNs (GFP-positive) over a monolayer of cortical astrocytes at 60 days post-infection. Notice the complex neuronal morphology; the yellow arrows highlight growth cone-like thickenings at the end of the extensions. (**B**–**B**″) Under these conditions, hmOEG-iNs (GFP-positive) express the neuronal markers Tuj1 and synapsin. (**C**–**C**′) Induced neurons also express ANK3, a specific marker of the axonal initial segment, proving the maturation status of hmOEG-iNs. **D** Quantification of GFP+ cells expressing the neuronal markers Tuj1, synapsin and ANK3 over the total of GFP+ cells, after transduction with NEUROD1-GFP. Scale bar: 100 µm (A), 50 µm (**C**, **C**′) and 10 µm (**B**–**B**″).

To enhance the functional maturation of hmOEG-iNs we co-cultured these cells over a monolayer of postnatal mouse astrocytes. After 60 dpi, we analyzed the culture by immunofluorescence and observed several GFP-positive cells acquiring a mature neuron morphology, with long axonal-like extensions, shorter dendritic-like extensions, and an enlargement at the end of some of the projections that could be associated with growth cones (Fig. 4A). Under these conditions hmOEG-iNs expressed the neuronal markers Tuj1, synapsin and, at the beginning of the axon, ANK3 (Fig. 4B–C′). ANK3 is a specific marker of the axonal initial segment and is required for the normal clustering of voltage-gated sodium channels at the axon hillock and for action potential firing, thereby proving the maturation status of hmOEG-iNs. The high percentage of positive cells (Fig. 4D) is the result of the death of those hmOEG-iNs that fail to reprogram to a mature state. Therefore, very few GFP+ cells will be found in culture, but those that have survived will express mature neuron markers.

**Fig. 5:**
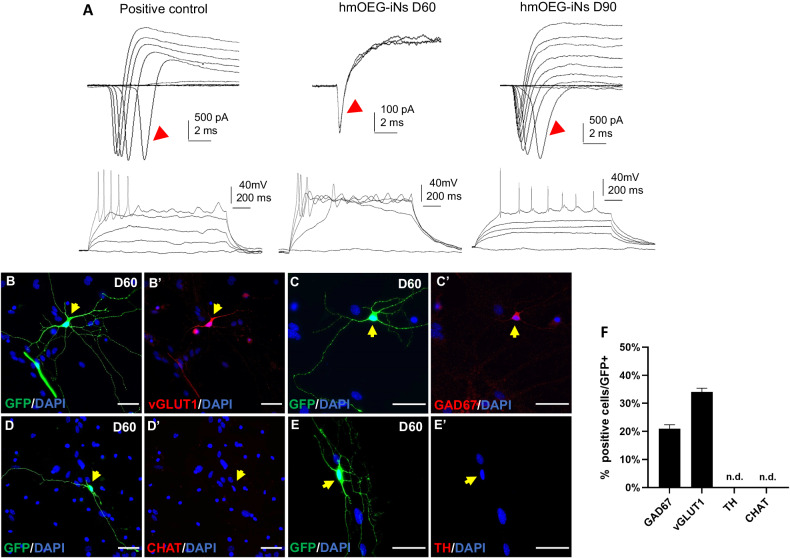
Functional maturation of hmOEG-iNs. **A** Electrophysiological recordings of a representative cortical neuron (left, as positive control), a representative neuron induced at 60 dpi (middle) and a representative neuron induced at 90 dpi (right). Notice that at 90 dpi, hmOEG-iNs have a sodium influx enough to fire a train of action potentials. Red arrows show the sodium channel currents. (**B**–**E**′) Immunofluorescence analysis of neuronal subtype markers: hmOEG-iNs expressed the glutamatergic neuron marker vGLUT1 (**B**, **B**′) and the GABAergic neuron marker GAD67 (**C**, **C**′), however, no cells stained positive for the markers CHAT (**D**, **D**′) or TH (**E**, **E**′), for cholinergic and dopaminergic neurons, respectively. Yellow arrows show hmOEG-iNs. **F** Quantification of the percentage of GFP+ cells expressing Gad67, vGlut1, CHAT or TH over the total of GFP+ cells. Scale bar: 50 µm. dpi days post-infection.

To determine the functionality of hmOEG-iNs, we performed electrophysiological assays using the patch-clamp technique. Recordings were performed on hmOEG-iNs identified with the GFP reporter, maintained in culture for 60 and 90 dpi. As a positive control we used embryonic mouse cortex neurons (Fig. 5A). Recording of hmOEG-iNs 60 dpi revealed a sodium current of 200 pA (200 ± 75 pA, *n* = 4) sufficient to trigger an action potential. At 90 dpi hmOEG-iNs showed a higher functional competence, recording an increase in sodium current up to 1000 pA (1000 ± 100 pA, *n* = 5) and firing a train of action potentials (5 ± 2 action potentials, *n* = 5) during the 1.5 s depolarizing current pulse (Fig. 5A).

The neuronal identity of hmOEG-iNs was assessed analyzing the expression pattern of different neuronal subtype markers. After 60 dpi, we detected hmOEG-iNs that expressed the glutamatergic neuron marker vGLUT1 (vesicular glutamate transporter 1) (Fig. 5B, B′, F) and GAD67 (glutamate decarboxylase 67) (Fig. 5C, C′, F), a GABAergic neuron marker. However, no cells stained positive for the markers CHAT (choline acetyltransferase) (Fig. 5D, D′, F), a marker specific for cholinergic neurons, or TH (tyrosine hydroxylase) (Fig. 5E, E′, F), a marker for dopaminergic neurons.

Taken together, these data indicate that ectopic expression of the single transcription factor NEUROD1 in hmOEG induces their direct conversion into glutamatergic and GABAergic functionally mature neurons.

### In vivo assay of hmOEG reprogramming

**Fig. 6:**
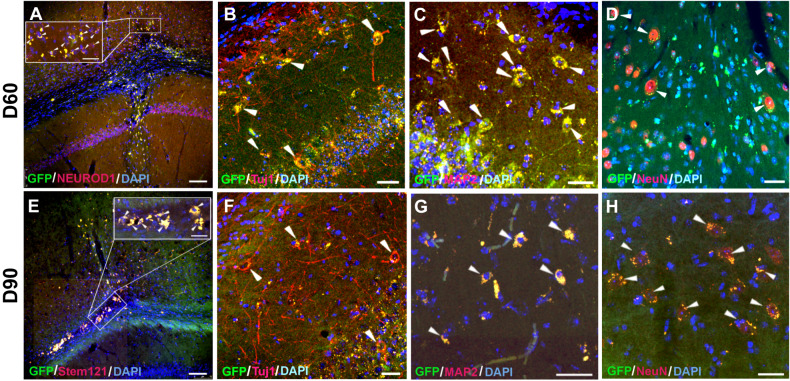
Transplantation of hmOEG-iNs into hippocampus of NOD-SCID mice. **A**, **E** Survival of hmOEG-iNs at 60 and 90 days post-transplant. GFP+ cells (green) co-express NEUROD1 (red; inset in **A**, arrowheads) or Stem 121 human antigen (red; inset in **E**, arrowheads), verifying the ectopic origin of these cells. (**B**–**D**, **F**–**H**) hmOEG-iNs survive within the brain parenchyma and stain positive for Tuj1 (red), MAP2 (red) and NeuN (red) neuronal markers (white arrowheads), suggesting conversion into neurons after transplantation. Nuclei were stained with DAPI (blue). Scale bar: 75 µm (**A**, **E**) and 25 µm (insets in **A**, **E**, **B**–**D**, **F**–**H**).

After achieving the direct conversion of hmOEG to functional neurons in vitro, we wished to verify that the committed reprogramming could take place in vivo. To do this, we infected the hmOEG primary cell line with the NEUROD1 factor and, one week after induction it was injected into the hippocampus of immunosuppressed NOD/SCID mice (detailed in “Methods”); two and three months after transplantation, we analyzed the ectopic hmOEG-iNs by immunofluorescence. In the injection site and deeper in the hippocampus we found GFP-NEUROD1-positive cells, of human origin (Stem121 human cell marker positive staining), along the time course (Fig. 6A, E). More in detail, some of these cells survived within the brain parenchyma and expressed the neuronal markers Tuj1 (Fig. 6B, F), MAP2 (Fig. 6C, G) and NeuN (Fig. 6D, H), suggesting that they might have been converted into neurons after transplantation. Unexpectedly, although NOD/SCID mice have a high degree of immunosuppression, a fraction of the ectopic hmOEG-NEUROD1 cell population was phagocytosed by microglia, in an environment of intense astroglial reactivity around the injection site (Supplementary Fig. S5A–C). These results show that after NEUROD1 lentiviral induction in vitro, hmOEG successfully integrate in brain tissue and neuronal fate commitment is maintained in an in vivo environment.

## Discussion

Several strategies have been used to overcome the scarce regenerative capacity of the CNS [46–48]. A recent approach is the conversion of somatic cells into pluripotent stem cells (iPSCs) and differentiate them into neurons for cell replacement therapy. However, iPSCs technology has suffered from some drawbacks as demonstrated by tumorigenesis, indeterminate differentiation or genomic instability [7, 8]. An alternative approach to bypass this downside is cellular direct conversion: to reprogram somatic cells into terminally differentiated neurons without going through a stem cell stage, following transplantation to the affected area [2–4]. In this work we directly reprogrammed olfactory ensheathing glia from adult human olfactory mucosa (hmOEG) to functional neurons, after transduction with the single neurogenic transcription factor NEUROD1, and verified that the committed reprogramming could take place in vivo after engraftment of transduced hmOEG cells.

An advantage of hmOEG over other cell types already reprogrammed to neurons is the reported capacity of OEG in promoting CNS regeneration [20]. OEG can be obtained from two sources, from the olfactory bulb and from the olfactory mucosa. No major differences in their neuroregenerative properties have been demonstrated to date [22, 49], although their genetic profiles have been shown to vary [50] and they promote differential axon sprouting with regard to the axonal tract, after transplantation in the injured spinal cord [27]. Therefore, with a view to possible clinical application, we chose hOEG from olfactory mucosa because a biopsy from the olfactory mucosa is less invasive than from the olfactory bulb, so autologous therapies could be performed and avoid post-transplant rejections. We evaluated hmOEG proregenerative capacity in co-cultures of hmOEG with axotomized adult rat retinal ganglion neurons (RGNs) and, both the percentage of RGNs extending axons and the mean axonal length/neuron of these axons increased significantly compared to the negative controls, proving the neuroregenerative properties of the starting cell line.

Most of glia-to-neuron conversion research has been carried out using virus-mediated ectopic expression of neurogenic transcription factors, on their own or in combination with other factors. [3, 5]. In our work, only ectopic expression of NEUROD1 induced the transition towards a neuronal phenotype. Consistent with our results, previous work shows the effectiveness of NEUROD1 inducing astrocyte to neuron reprogramming [14, 15, 51]. However, our results are not in agreement with those previously published by Sun et al. [52] which demonstrated that OEG from adult mice can be directly reprogrammed into neuronal cells by the transcription factor NEUROG2; moreover, these authors reported that NEUROD1 expression did not result in neuronal conversion. This discordance could be due to a different mechanism inducing cell reprogramming between both species. Thus, RNA expression diversity between humans and mice [53], could provide different contexts, in terms of endogenous transcriptional networks and the chromatin state of the starting cell, affecting the reprogramming cell fate [2, 54].

A concerning issue was the low reprogramming efficiency after 60–90 dpi, which was accompanied by the death of those cells that did not make it to the iN fate. This effect could be attributed to the metabolic shift during the conversion process, increasing the production of reactive oxygen species (ROS) and inducing cell death by oxidative stress [55]. Another process suggested to hinder direct reprogramming is genomic stress, which is caused by interference between high levels of transcription and replication, resulting in high cell death [56]. Therefore, induction will occur in most cells, but few cells would be fully reprogrammed. Additionally, the starting cell type conversion efficiency will depend on transcriptional accessibility of target genes in a permissive epigenetic environment. Therefore, co-factors that remove epigenetic barriers to reprogramming (e.g. repressive DNA methylation and histone modifications) can enable a neuronal gene expression program and improve transcription factor-mediated neuronal direct conversion [6].

Under appropriate culture conditions, neuronal maturation was achieved, obtaining electrophysiologically competent neurons which expressed glutamatergic or GABAergic markers. It is unclear whether a specific neuronal subtype can be preferentially induced from direct reprogramming of heterologous cells. In previous works, overexpression of NEUROD1 resulted in glutamatergic or, to a lesser extent, GABAergic neurons, starting from astrocytes, NG2 glia or microglia [15, 17]. Likewise, induction of mouse OEG reprogramming using NEUROG2 resulted in a mixed population of glutamatergic and GABAergic neurons [52]. In addition, it has been possible to direct cell reprogramming towards a particular neuronal subtype by adding transcription factors involved in cell specification during neuronal development. Thus, reprogramming of striatal astrocytes to GABAergic neurons was induced by a combination of NEUROD1 and DLX2 [16].

A reported feature of direct conversion is the lack of stemness of the induced somatic cell population along the reprogramming process. Thus, functional neurons from fibroblasts, astrocytes or microglia have been generated without reverting to a progenitor cell stage [10, 13, 17]. However, some authors have reported that cells in the reprogramming trajectory could pass through a neural stem cell-like state, prior to differentiating into iNs [44, 45, 57]. This dedifferentiated stage is characterized by transient expression of genes that are normally expressed in neural stem or progenitor cells during embryonic development. After the analysis of SOX2 expression, a characteristic marker of neuronal precursors during the first days of induction, and of the cell proliferation marker KI67, our results suggested that the reprogramming process did not go through a proliferative dedifferentiation stage. Such intermediate, stem cell-like state was not detected either in murine OEG to neuron direct conversion [52].

Induced neurons (iNs) generated in vitro can be subsequently transplanted at the site of the lesion [6]. After achieving the direct conversion of hmOEG to functional neurons in vitro, we engrafted transduced hmOEG cells in mouse hippocampus: these cells showed specific neuronal labeling, suggesting their conversion to neuronal cells after transplantation. Although we cannot completely exclude that any of the GFP-positive neurons could be the result of a fusion event with endogenous cells, the distribution of the human markers makes us rule out that this is a widespread event. However, a fraction of the ectopic hmOEG-NEUROD1 cell population was phagocytosed by microglia, in an environment of intense astroglial reactivity around the injection site, despite the use of NOD-SCID immunosuppressed mice. Although unexpected, rejection of autologous iPSCs grafts has already been reported [58] and this immune response has been suggested to be triggered by epigenetic alterations present in the reprogramming cell lines [59].

In conclusion, in this work we succeeded in directly converting olfactory ensheathing glia from adult human olfactory mucosa (hmOEG) to neurons. We showed that the cells under study maintained the characteristic neuroregenerative properties of OEG and, after transduction of the single neurogenic transcription factor NEUROD1, they exhibited morphological and immunolabeling neuronal features, fired action potentials and expressed glutamatergic and GABAergic markers. In addition, after engraftment of transduced hmOEG cells in mouse hippocampus, these cells showed specific neuronal labeling. Thereby, if we add to the neuroregenerative capacity of OEG cultures the conversion to neurons of a fraction of their population through reprogramming techniques, the engraftment of OEG and OEG induced neurons (OEG-iNs) could be a procedure to enhance neural repair after central nervous system injury.

### Supplementary information

Table 1

Supplementary Figure S1. Characterization of hmOEG cell line: analysis of OEG markers.

Supplementary Figure S2. Characterization of hmOEG cell line: analysis of neuronal markers.

Supplementary Figure S3. Screening of transcription factors to convert hmOEG to induced neurons (hmOEG-iNs).

Supplementary Figure S4: NEUROD1-hmOEG does not transition to a neural stem cell-like state before differentiating into iNs.

Supplementary Figure S5: Reactivity after hmOEG-iNs transplantation into the brain of NOD-SCID mice.

## Data Availability

All data generated or analyzed during this study are included in this article and its Supplementary Information files.
