# Supplementary material for: Generation of functional neurons from adult human mucosal olfactory ensheathing glia by direct lineage conversion
Source: Cell Death Dis. 2024 Jul 3;15(7):478. doi: 10.1038/s41419-024-06862-9 (PMC11222439; doi:10.1038/s41419-024-06862-9)
Supplement: Supplementary file 2 — Supplementary Figure S1. Characterization of hmOEG cell line: analysis of OEG markers. [file 41419_2024_6862_MOESM2_ESM.pptx]

## Slide 1
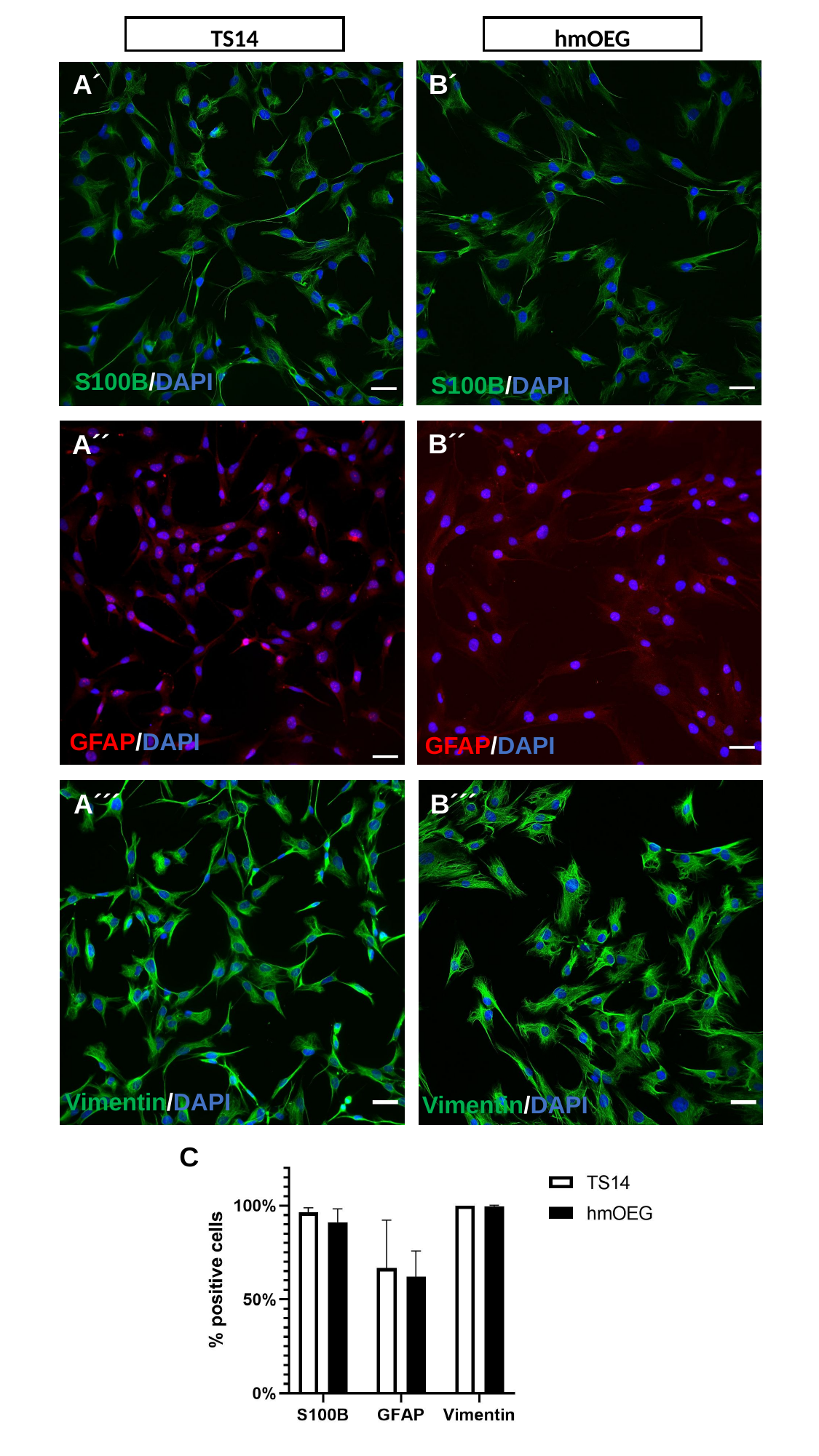

TS14
hmOEG
A´
B´
S100B/DAPI
S100B/DAPI
B´´
A´´
GFAP/DAPI
GFAP/DAPI
B´´´
A´´´
Vimentin/DAPI
Vimentin/DAPI
C

## Slide 2
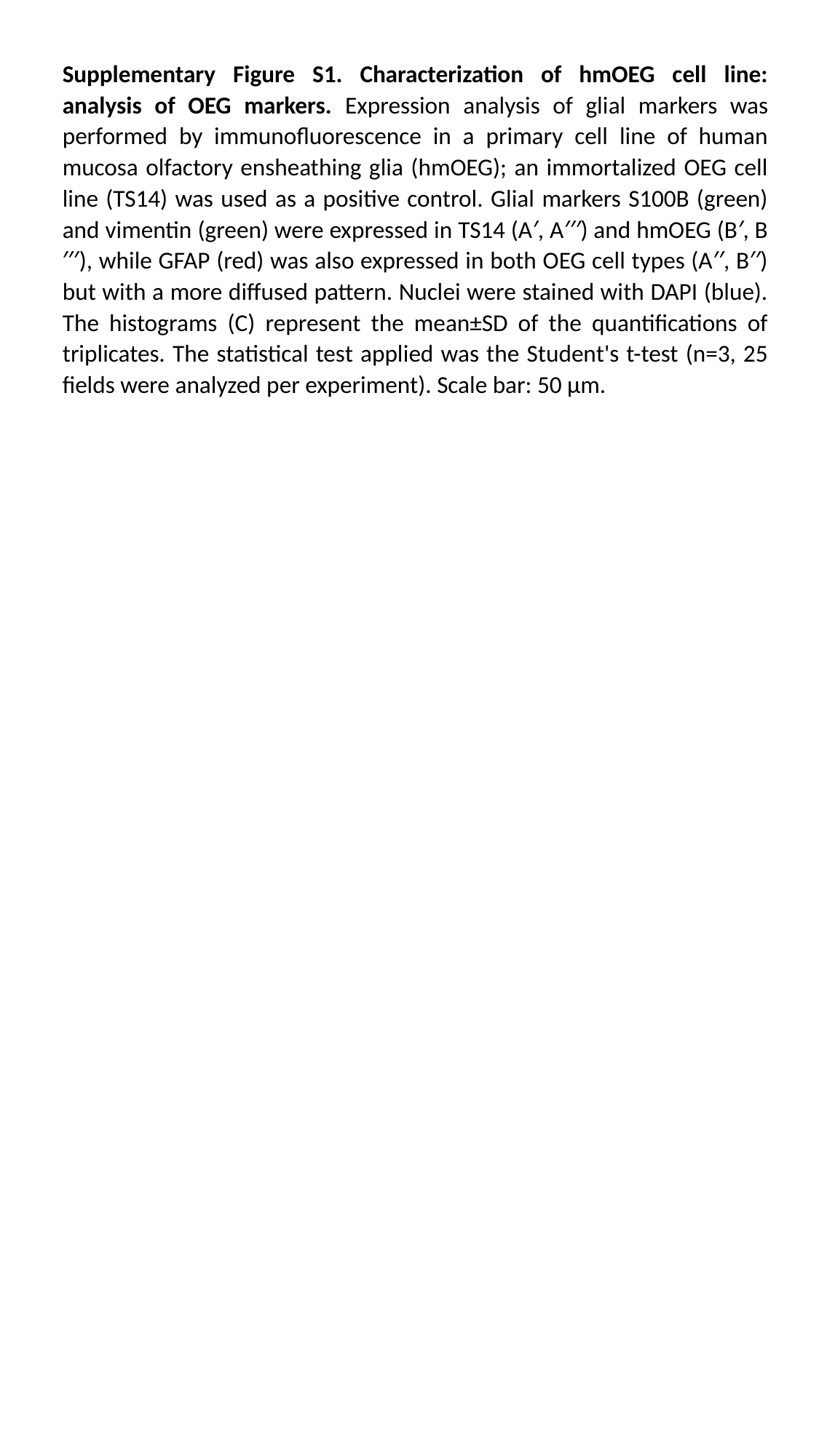

Supplementary Figure S1. Characterization of hmOEG cell line: analysis of OEG markers. Expression analysis of glial markers was performed by immunofluorescence in a primary cell line of human mucosa olfactory ensheathing glia (hmOEG); an immortalized OEG cell line (TS14) was used as a positive control. Glial markers S100B (green) and vimentin (green) were expressed in TS14 (A′, A′′′) and hmOEG (B′, B′′′), while GFAP (red) was also expressed in both OEG cell types (A′′, B′′) but with a more diffused pattern. Nuclei were stained with DAPI (blue). The histograms (C) represent the mean±SD of the quantifications of triplicates. The statistical test applied was the Student's t-test (n=3, 25 fields were analyzed per experiment). Scale bar: 50 µm.
