# Supplementary material for: Generation of functional neurons from adult human mucosal olfactory ensheathing glia by direct lineage conversion
Source: Cell Death Dis. 2024 Jul 3;15(7):478. doi: 10.1038/s41419-024-06862-9 (PMC11222439; doi:10.1038/s41419-024-06862-9)
Supplement: Supplementary file 4 — Supplementary Figure S3. Screening of transcription factors to convert hmOEG to induced neurons (hmOEG-iNs). [file 41419_2024_6862_MOESM4_ESM.pptx]

## Slide 1
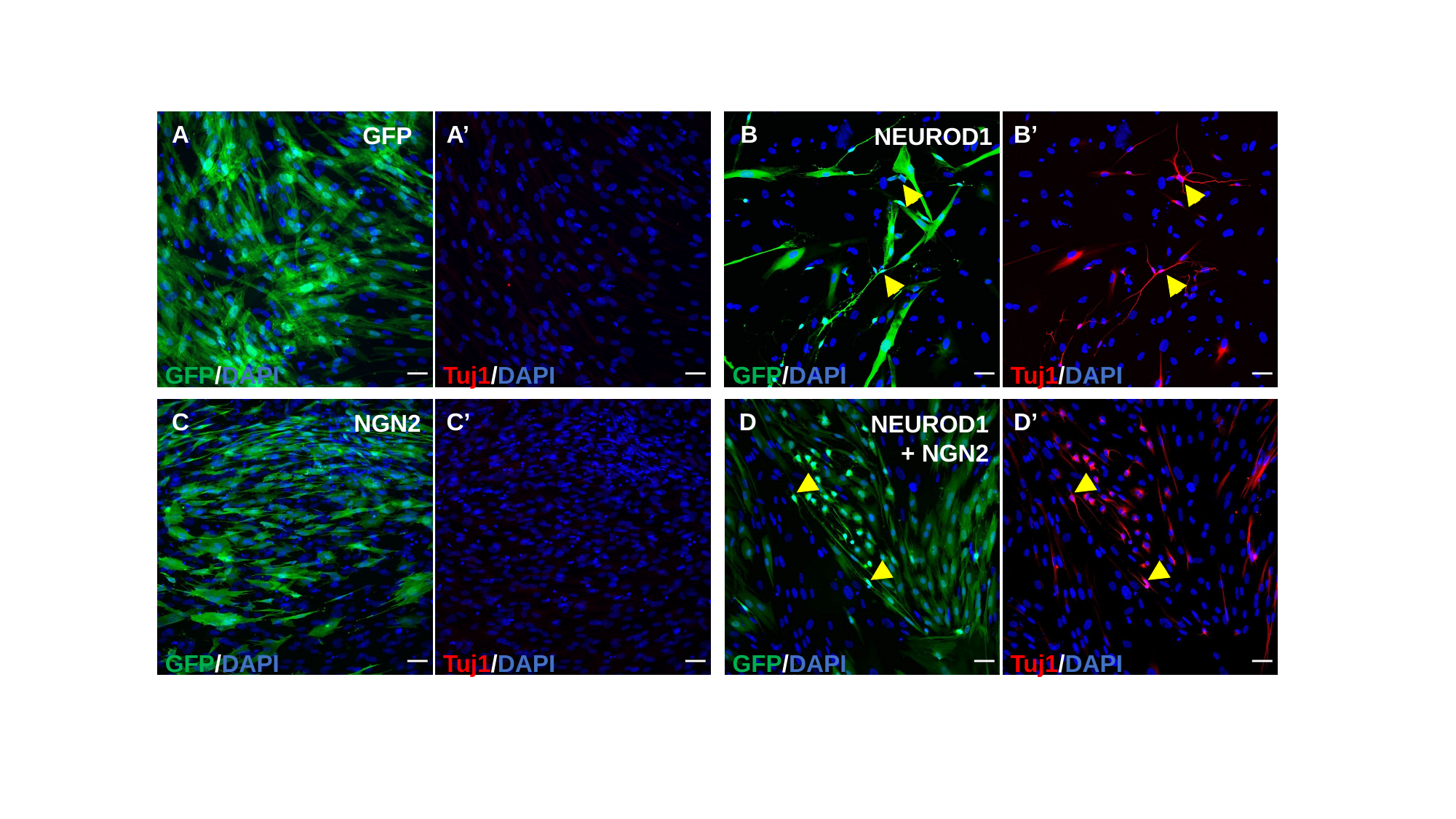

A
A’
B
B’
GFP
NEUROD1
GFP/DAPI
Tuj1/DAPI
GFP/DAPI
Tuj1/DAPI
C
C’
D
D’
NGN2
NEUROD1
+ NGN2
GFP/DAPI
Tuj1/DAPI
GFP/DAPI
Tuj1/DAPI

## Slide 2
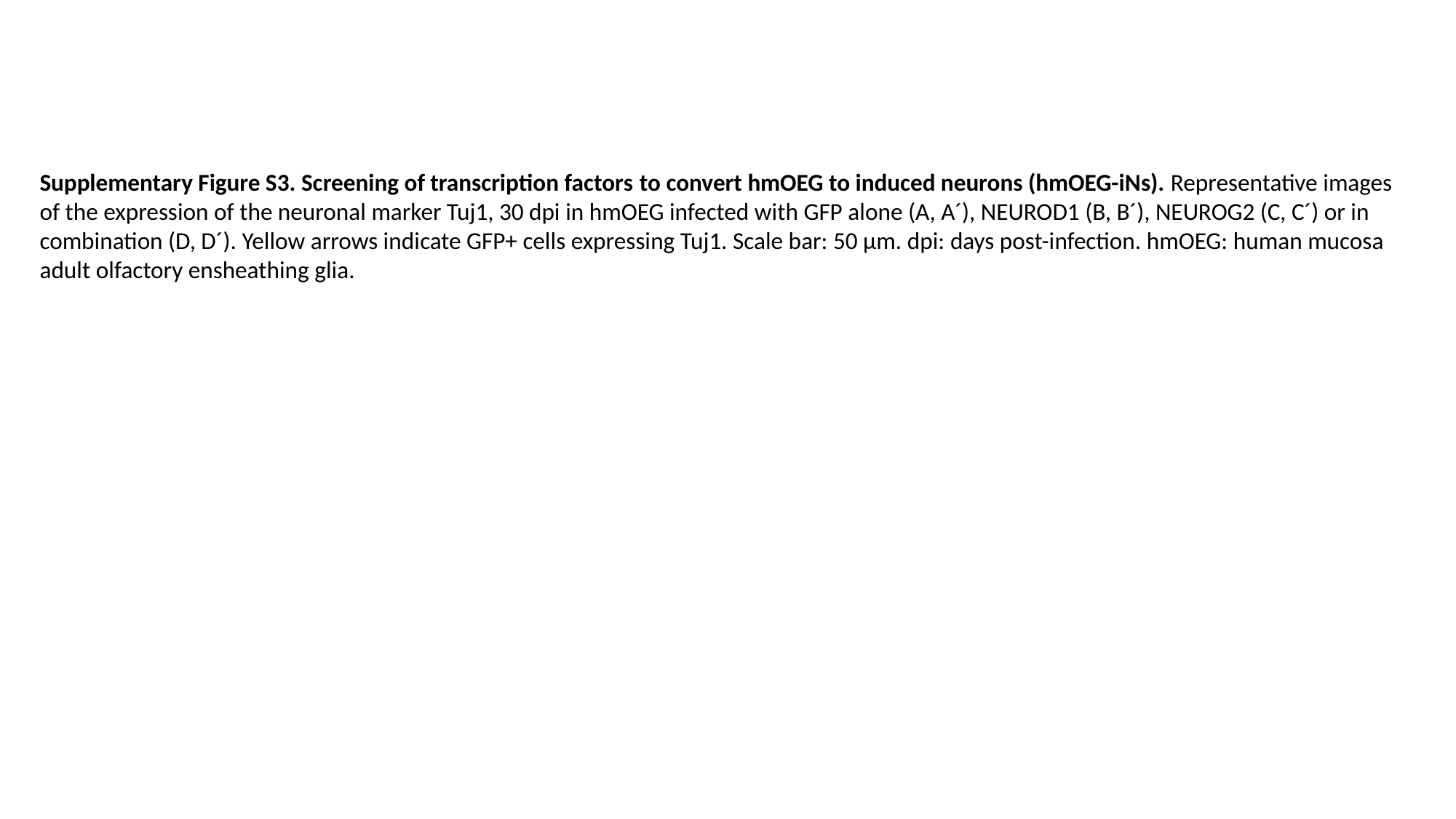

Supplementary Figure S3. Screening of transcription factors to convert hmOEG to induced neurons (hmOEG-iNs). Representative images of the expression of the neuronal marker Tuj1, 30 dpi in hmOEG infected with GFP alone (A, A´), NEUROD1 (B, B´), NEUROG2 (C, C´) or in combination (D, D´). Yellow arrows indicate GFP+ cells expressing Tuj1. Scale bar: 50 µm. dpi: days post-infection. hmOEG: human mucosa adult olfactory ensheathing glia.
