# Supplementary material for: Generation of functional neurons from adult human mucosal olfactory ensheathing glia by direct lineage conversion
Source: Cell Death Dis. 2024 Jul 3;15(7):478. doi: 10.1038/s41419-024-06862-9 (PMC11222439; doi:10.1038/s41419-024-06862-9)
Supplement: Supplementary file 5 — Supplementary Figure S4: NEUROD1-hmOEG does not transition to a neural stem cell-like state before differentiating into iNs. [file 41419_2024_6862_MOESM5_ESM.pptx]

## Slide 1
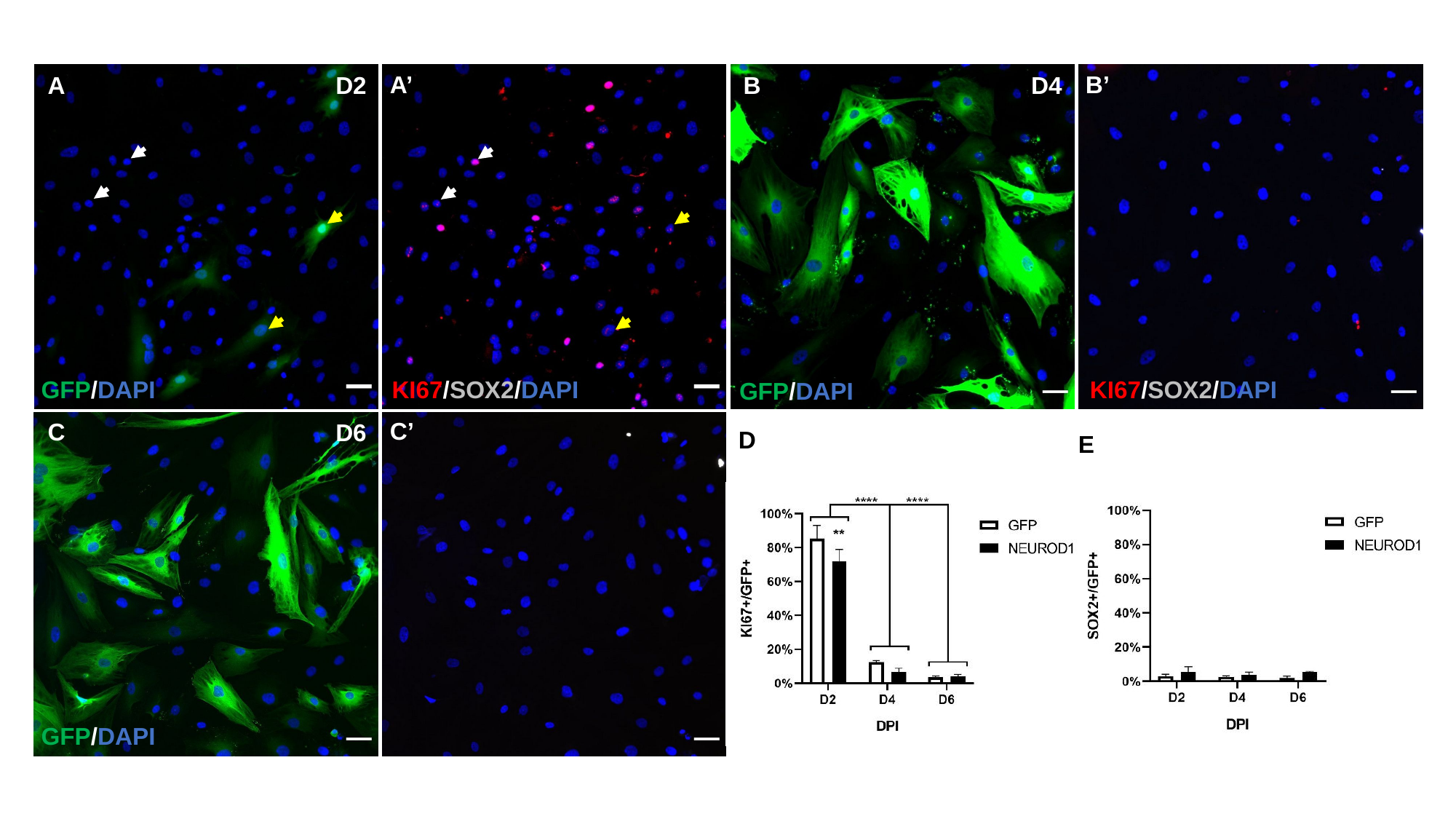

A’
B’
A
B
D2
D4
KI67/SOX2/DAPI
KI67/SOX2/DAPI
GFP/DAPI
GFP/DAPI
C’
C
D6
D
E
GFP/DAPI
AAAA

## Slide 2
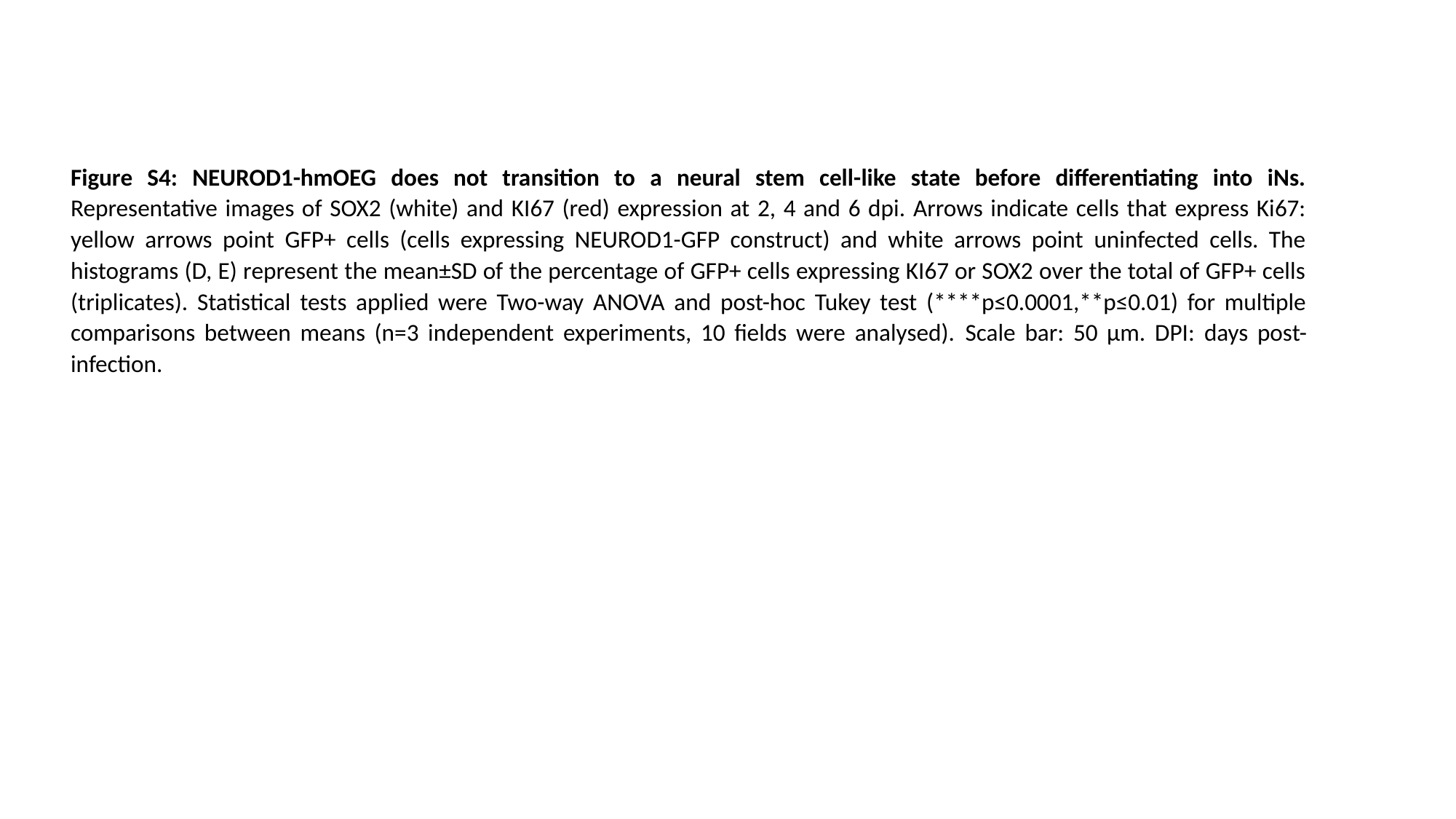

Figure S4: NEUROD1-hmOEG does not transition to a neural stem cell-like state before differentiating into iNs. Representative images of SOX2 (white) and KI67 (red) expression at 2, 4 and 6 dpi. Arrows indicate cells that express Ki67: yellow arrows point GFP+ cells (cells expressing NEUROD1-GFP construct) and white arrows point uninfected cells. The histograms (D, E) represent the mean±SD of the percentage of GFP+ cells expressing KI67 or SOX2 over the total of GFP+ cells (triplicates). Statistical tests applied were Two-way ANOVA and post-hoc Tukey test (****p≤0.0001,**p≤0.01) for multiple comparisons between means (n=3 independent experiments, 10 fields were analysed). Scale bar: 50 µm. DPI: days post-infection.
