# Supplementary material for: Generation of functional neurons from adult human mucosal olfactory ensheathing glia by direct lineage conversion
Source: Cell Death Dis. 2024 Jul 3;15(7):478. doi: 10.1038/s41419-024-06862-9 (PMC11222439; doi:10.1038/s41419-024-06862-9)
Supplement: Supplementary file 6 — Supplementary Figure S5: Reactivity after hmOEG-iNs transplantation into the brain of NOD-SCID mice. [file 41419_2024_6862_MOESM6_ESM.pptx]

## Slide 1
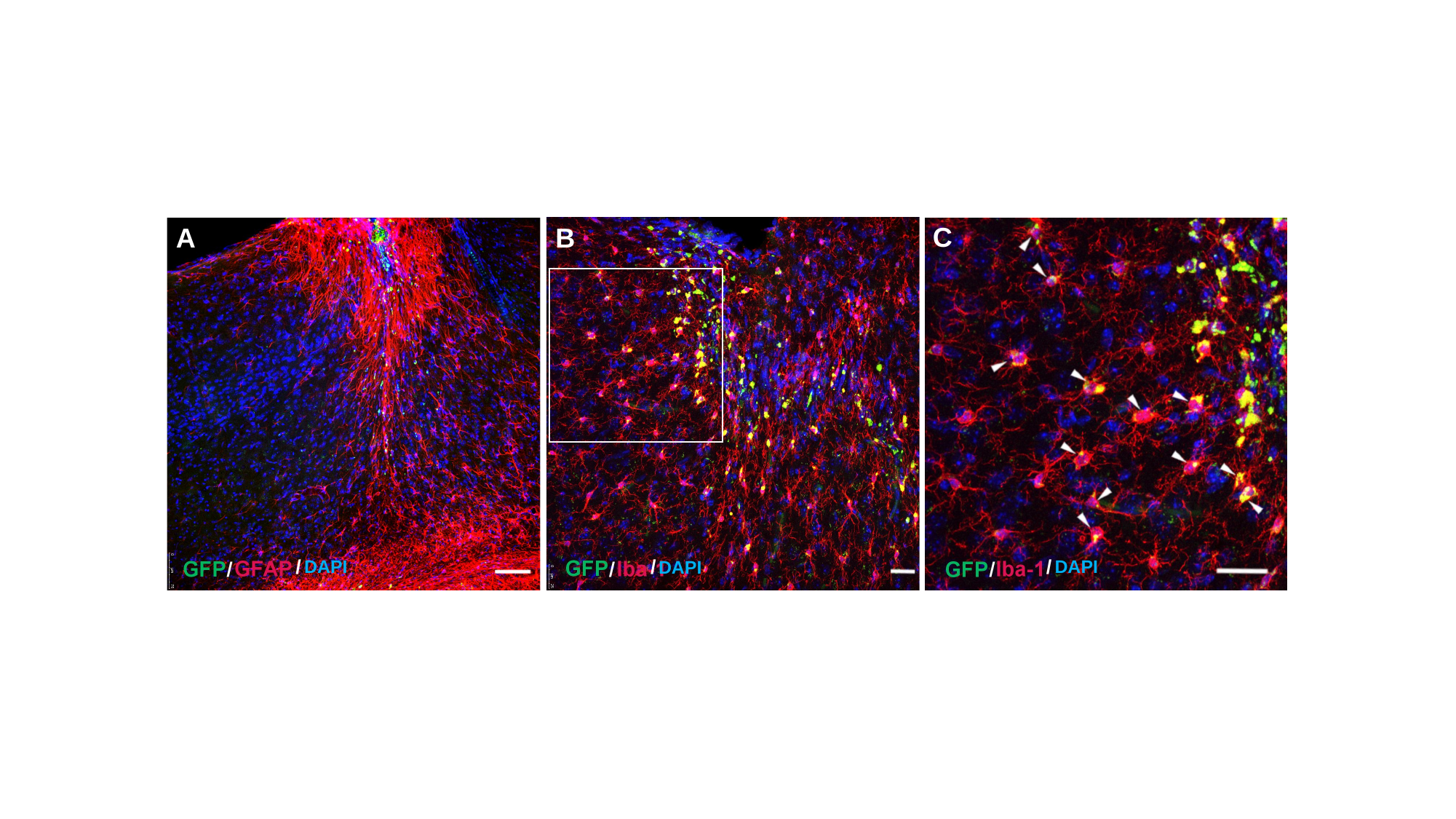

C
A
B
/
/
/
DAPI
DAPI
DAPI

## Slide 2
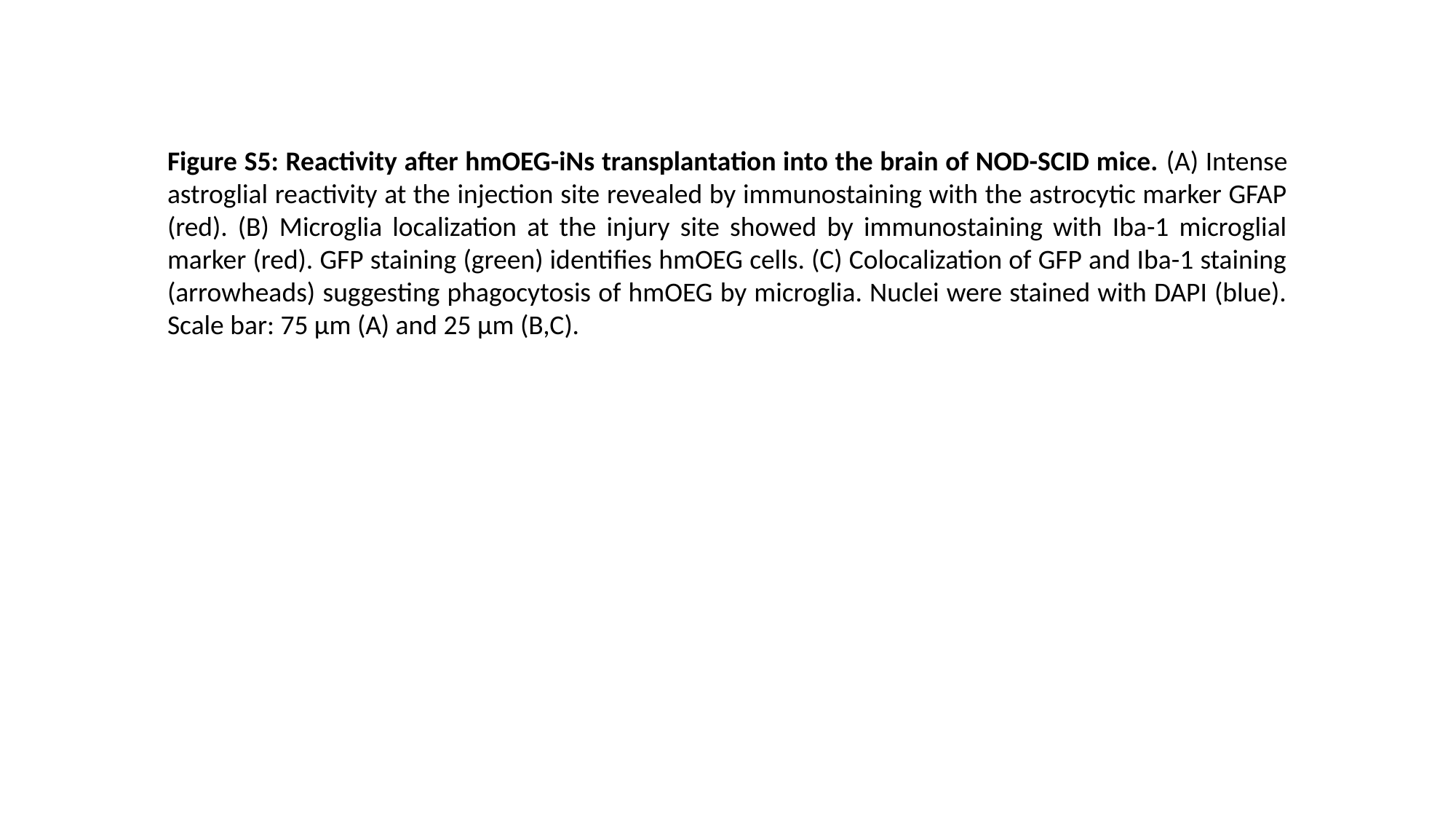

Figure S5: Reactivity after hmOEG-iNs transplantation into the brain of NOD-SCID mice. (A) Intense astroglial reactivity at the injection site revealed by immunostaining with the astrocytic marker GFAP (red). (B) Microglia localization at the injury site showed by immunostaining with Iba-1 microglial marker (red). GFP staining (green) identifies hmOEG cells. (C) Colocalization of GFP and Iba-1 staining (arrowheads) suggesting phagocytosis of hmOEG by microglia. Nuclei were stained with DAPI (blue). Scale bar: 75 µm (A) and 25 µm (B,C).
